# Zeolites at the Molecular Level: What Can Be Learned from Molecular Modeling

**DOI:** 10.3390/molecules26061511

**Published:** 2021-03-10

**Authors:** Ewa Broclawik, Paweł Kozyra, Mariusz Mitoraj, Mariusz Radoń, Paweł Rejmak

**Affiliations:** 1 Jerzy Haber Institute of Catalysis PAS, Niezapominajek 8, 30-239 Krakow, Poland; 2 Faculty of Chemistry, Jagiellonian University, Gronostajowa 2, 30-387 Krakow, Poland; kozyra@chemia.uj.edu.pl (P.K.); mitoraj@chemia.uj.edu.pl (M.M.); mradon@chemia.uj.edu.pl (M.R.); 3 Laboratory of X-ray and Electron Microscopy Research, Institute of Physics Polish Academy of Sciences, Al. Lotnikow 32/46, 02-668 Warsaw, Poland; rejmak@ifpan.edu.pl

**Keywords:** molecular modeling, zeolite, transition metal sites, Brønsted and Lewis acid sites, multiple bond activation, DFT, wave function methods, electron density distribution

## Abstract

This review puts the development of molecular modeling methods in the context of their applications to zeolitic active sites. We attempt to highlight the utmost necessity of close cooperation between theory and experiment, resulting both in advances in computational methods and in progress in experimental techniques.

## 1. Introduction

**Figure 1:**
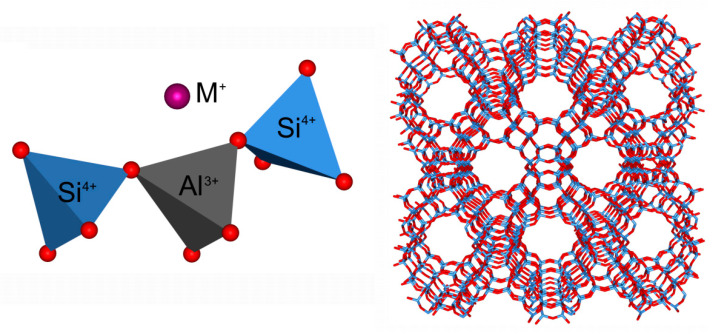
The basics of the structural chemistry of zeolites explained: zeolites are crystalline, microporous tectoaluminosilicates, wherein Si and Al atoms, collectively referred to as ‘T atoms’, are tetrahedrally coordinated by O atoms. These TO_4_ units, connected by their apices (i.e., O atoms), form a three-dimensional network (left panel). Viewing the lattice as an ionic solid, the replacement of Si^4+^ ions by Al^3+^ ones creates an excess of negative charge, which is compensated by extraframework cations M^+^ (e.g., H^+^, NH_4_^+^, metal cations).

Catalysts based on microporous and hierarchical zeolitic materials are the subject of extensive academic and industrial research. Their long-lasting topicality stems from the many distinctive features of these materials, such as their high surface area, high hydrothermal and thermal stability, and the presence of the Brønsted and Lewis acid sites of controlled strength. Figure 1 schematically depicts the basics of the structural chemistry of zeolites, which are crystalline, microporous tectoaluminosilicates, wherein Si and Al atoms (collectively referred to as ‘T atoms’) are tetrahedrally coordinated by O atoms.

Substitution of Si by Al in the aluminosilicate framework leads to the presence of Brønsted acid sites, leading to a wide range of applications including, among others, in major branches of petrochemical industry, where the production of hydrocarbons through the steam cracking of fossil-based feedstock relies on the Brønsted acidity of zeolites. In turn, Brønsted protons replaced (in the absence of water) by ion-exchanged cations may form Lewis acid or basic sites, which opens up novel catalytic applications. Furthermore, transition metal ions (TMI) positioned at these sites may be coordinatively unsaturated and can switch between various oxidation states, generating subsequent reactivity as redox catalysts and opening up new branches of application in fine chemistry and redox catalysis. TMI-zeolites are very complex catalysts due to the intrinsic properties of TMI, to frequent imperfections (crystal defects, silanol groups, extraframework aluminum), and to admixture of other cations (commonly H^+^, TMI, Na^+^, K^+^, Ca^2+^, Mg^2+^). Moreover, the crystallographic distribution of aluminum is sparsely detailed. As a consequence, the comparison of data published by various research groups is challenging, and the molecular structure of active sites and their role in the catalytic mechanism is difficult to discern. Although the structure of Brønsted site in zeolites seems much better defined as bridging hydroxyls with characteristic O–H stretching frequencies and NMR signals, their impact on the overall catalyst activity is also intricate and relies on many additional factors; thus, it cannot be framed within simple correlation relations. All told, independent tools such as molecular modeling are needed to corroborate modern experiments in unraveling the complicated nature of zeolite catalysis.

In this contribution, we shall outline open challenges in the field of molecular modeling, with the stress put on our own experience with applying various modeling methodologies to zeolitic active sites and their catalytic performance. The reader interested in general aspects of zeolite chemistry and their applications is referred to the comprehensive review on state of the art and future challenges of zeolites as catalysts [1] and to topical reviews on specific applications of zeolites in oil refining [2], in sustainable chemistry [3], and in biotechnology and medicine [4]. In turn, a general introduction to recent advances in atomistic simulation methods, the development of material databases and the use of machine learning for the prediction of zeolite properties can be found in many excellent up-to-date reviews [5,6,7,8,9].

Our story opens with early simplistic modeling of straightforward properties of zeolitic active sites to assist profound interpretation of experimental research triggered by the discovery of exceptional activity of copper(I) site in Y (a high-silica type of faujazite) and ZSM-5 (Zeolite Socony Mobil–5, of MFI type) zeolites towards NO decomposition (Section 2). Since its publication by Iwamoto [10,11] this catalytic process has raised scientists’ hopes and inspired researchers toward intense studies via experimental [12,13,14,15] as well as via computational methods [16,17,18,19,20,21]. In due time, the vast increase in accessible computer resources and stunning development of expert numerical routines extended tractable molecular models to giant sizes, with further extension to a variety of embedding schemes without the loss of direct auxiliary techniques such as refined geometry optimization, second derivatives, etc. Additionally, the application of periodic boundary conditions to solids with large supercells needed to catch real conditions in catalysis became feasible [22]. Thus, the following sections will deal with selected examples of mixed QM/MM and periodic studies on catalytic active site in zeolites (Section 3).

Along with the development of reliable models of catalytic active sites at a molecular level, newly developed quantum chemical methods and tools helped to increase the accuracy of electronic structure and to extract additional information from electron density, along with its deformation upon mutual interactions in multi-component system (zeolite framework, catalytic center and the substrate). The contribution of this type of post-analysis to the knowledge regarding zeolite catalysts will be discussed in separate sections (Section 4 and Section 5).

Topics adduced so far in the context of the molecular modeling of zeolites have addressed selected pitfalls and challenges stemming from the need to define limited molecular models (tractable by computation techniques with acceptable accuracy) for the active site at a solid surface. When it comes to transition metal sites (such as Fe or Co), successive difficulties emerge, stemming from the electronic structure of the center per se. These involve strong correlation, variability of oxidation states and spin states as well as significant coupling between electronic and geometric degrees of freedom. When the issue of non-innocent (strongly interacting) ligands is added, it becomes clear that the advanced correlated electronic structure methods must come into play. Thus, the next section will be devoted to spin-resolved methods and wave function techniques in view of their dedicated applications to zeolites (Section 5).

In the final section, some remarks will be given on computational determination of entropy and reactive free-energy surfaces, with the stress put on credibility of predictions and consequences for qualitative insights into heterogeneous catalysis. Here, ab initio molecular dynamics (AIMD) simulations will be briefly discussed in the context of simulating realistic vibrational frequencies and explicit temperature behavior of catalytic systems.

## 2. Interplay between Experiment and Theory in Describing the Catalytic Properties of Metal Active Site in Zeolites

As already alluded to, we hope to illustrate (mostly on the basis of our own experience) how the broadening of the spectrum of properties accessible from an experimental base has mutually inspired the development of computational tools for extracting information deeply embedded in the raw results of molecular modeling. Not only the advanced properties could be approached with increasing accuracy through the use of smart computational protocols, but deeper understanding and guidance might also be gained on the basis of hi-tech experimentation. Concise methodological remarks concerning molecular modeling methods will be given in Section 3.1; here, we briefly summarize early attempts to model Bronsted and Lewis acid/base site in zeolites. At that time, the gradual emergence of practical implementations of *first principle* or *semiempirical* molecular orbital theory schemes using small molecular models opened a new field of research, i.e., simulations for catalysis, despite access to sufficient computer resources and highly effective quantum chemical routines still being limited [23]. Our original contributions started with the attempt to reproduce spectroscopic NMR and the infrared signatures of bridging hydroxyls by applying semiempirical molecular orbital theory to the clusters (AlO)_(n−1)_(SiO)_(4−n)_[Si-OH-Al](OSi)_3_, with n = 1–4, mimicking the zeolitic environment of four types of bridging hydroxyls, suggested in the faujazite framework [24,25]. We found good correlation between the calculated properties and the spectroscopic IR and NMR data; however, it must be recalled here that such simplistic modeling could only be reliably trusted after extensive experimental validation.

In the subsequent decades, the continuous advances made in computing power and software development dramatically changed the status of molecular modeling. State-of-the-art calculations on massively parallel machines continually enlarged the scope of what was accessible with improved methodologies. This triggered the outburst of theoretical studies on zeolite catalysis [26]. The next large step forward was made with robust implementations of density functional theory (DFT), enabling a practical approach to catalytic reaction pathways and reaction mechanisms, even though exchange transition metals were involved [27]. Our studies on the catalytic reaction mechanism in Ga-exchanged zeolites were among the first contributions in this field. Density functional theory was used to describe the reaction profile for the initial steps of methane dissociation on Ga-exchanged ZSM-5, where stable structures on the reaction pathway were characterized and the transition state was explicitly defined [28,29].

Nevertheless, constructing appropriate, although rather arbitrary, structural models of zeolitic sites was indispensable, since experimental evidence on Al distribution in the silicalite framework and the exact positions of exchanged cations was still limited. On the other hand, the most robust computational tools had been commercialized and were not widely available for scholarly research. In this spirit, six-ring or five-ring clusters in the vicinity of the T12 or T8 framework positions (T site—silicon or aluminum node in zeolite lattice) with one or two aluminum atoms were proposed as appropriate models to accommodate Cu^+^ and Cu^2+^ cations, respectively [30]. The Si-O-Al rings (optimized with and without the cation) were then superimposed, and the mean square root deviations between coordinates of atoms in the ring were calculated. They were taken as signatures of the strength of the framework interaction with the hosted cation, and successfully related to the shifts of frequencies of skeletal vibrations (indirect IR measure of the interaction strength). In this way, the information on skeletal modes was extracted from the small cluster calculations, evidencing that simplistic models, when used with care, are capable of mimicking the inherent global properties of the framework, such as its distortion upon copper substitution.

Encouraged by this promising perspective and the growing importance of zeolite catalysts in the environmental sciences, we applied a similar computational protocol in close cooperation with IR data to selected metal sites in zeolites. Special attention was paid to the interaction of probe molecules with the metal center, serving both as an experimental tool for testing the properties of the active site and as a target for catalysis. In the years 2000–2006, a series of studies based on small cluster models of zeolite environments were undertaken to investigate the cationic site in zeolites (mainly Cu^+^ and Cu^2+^, occasionally other cations like Na^+^ or Co^2+^) and their interaction with small ligands [21,31,32,33,34,35,36,37]. Molecules, which were simultaneously studied by IR spectroscopy, such as standard probe-molecules (N_2_, CO and nitriles), inorganic molecules of reactants (NO, H_2_) and organic molecules with *π* electrons (alkenes, alkynes, ketones) were subjected to the analysis. The main advantage of the series was the simultaneous correlation of the molecular modelling results with the IR experiment, serving as a source of information about the heterogeneity and accessibility of these sites in various catalytic processes in zeolites [38,39,40,41,42,43].

Extensive experimental verification was critical for the assessment of the data obtained in silico by means of simplistic modeling. Therefore, diverse factors related with the property of interest were examined, for example, the values of bond elongation (∆*r,* calculated) and the changes of wavenumber for stretching vibration upon interaction with respect to the gas phase molecule (∆*ν*, measured and calculated) were used as the measures of the extent of bond activation. Both ∆*r* and ∆*ν* vary significantly among the molecules interacting with the discussed cations (by up to two orders of magnitude), and they were compared with experimental data from our own resources for the flagship case of Cu(I)ZSM-5 [34,35,36,37,38,39,40,41,42,43]. The type of model chosen could be rationalized and used as a predictive tool for other properties and related systems only after a sufficient set of diverse factors characterizing the site showed satisfactory agreement with the experiment.

At that time, the deNOx aspect of copper exchanged zeolite catalysts seemed to be the most attractive, and thus cationic site models (already tested at the available level of theory, vide infra) were applied to the analysis of the interaction between the site and the possible reactants. Certainly, the agreement between the calculated and measured IR frequencies or adsorption energies was satisfactory, rather than perfect, due to the numerous limitations of the molecular modeling tools. As an example, let us discuss the case of NO adsorbed on the Cu(I) site in Cu-ZSM-5: the IR spectrum exhibits a red-shift of the NO stretching frequency by −67 cm^−1^, while upon adsorption onto the Cu(II) site, the frequency increases by +29 cm^−1^ [21]. Our calculations yielded red-shifts of −106 and −34 cm^−1^, respectively, which was far from satisfactory. However, it must be recalled here that the best available result published in 2004 (obtained using an upgraded QM/MM methodology, making it possible to discern various zeolitic sites) was the shift by −117 cm^−1^ for NO adsorbed on Cu(I)MFI and by −108 cm^−1^ for NO on Cu(I) ferrierite [44], comparable to our results. The difficulty of the exact reproduction of NO stretching frequencies has been attributed to several factors, including strong coupling to the bending mode, anharmonic contributions, or lack of temperature effects in static quantum chemical calculations, and these have not been definitely solved, as yet. On the other hand, better reproduction could be expected for relative values representing physicochemical observables (due to the profitable cancelation of errors); indeed, the difference between the shift of NO frequency caused by the interaction with the Cu(I) vs. the Cu(II) site in the same zeolite remained within acceptable limits: 96 cm^−1^ (measured) and 72 cm^−1^ (calculated), respectively [21].

Therefore, our temporary goal was not the exact reproduction of experimental data, but rather the thorough exploration of the rational causes and consequences of the studied phenomena, in this case, the physicochemical background behind the activation of the NO bond and its consequences for catalysis. The following examples should give some flavor of the subsequent work devoted to the activation of hydrocarbons and selected inorganic ligands caused by their adsorption onto copper, cobalt or other metal cations and, last but not least, Bronsted acid site in zeolites [45,46,47,48,49,50].

The study of the activation of ethene and ethyne by Cu(I) and Ag(I) site In ZSM5 zeolite should provide a good example of the extreme caution necessary for the credible interpretation of computational results. Our calculations indicated the weakening of the carbon-carbon bond, evidenced by the CC bond elongation as well as the lowering of the frequency of the corresponding stretching vibrations [42,43,51]. Qualitative agreement with the experimental data was obtained for both hydrocarbons: on both cations, the calculated and measured red-shifts of CC frequency for ethyne were far greater than those for ethene, while both were significantly lower for silver than for copper sites. However, quantitative comparison was far from perfect, and some implications stemming from the observed trends seemed counterintuitive: although the calculated charge transferred from the metal center to the adsorbed molecules for the Ag(I) site exceeded that for the Cu(I) site, the weakening of the CC multiple bond at the Ag(I) site was substantially less pronounced (as evidenced by both calculations and experimentation). Only after additional analysis of the components of the electron density flow between selected fragments and diligent assessment of their role in the activation process could some general conclusions be reached based on electronic properties [52,53,54]. This topic and other insights into electronic aspects behind the activation of various ligands by cationic sites in zeolites resulting from the analysis of electron density reorganization upon adsorption will be discussed in conjunction with the development of novel tools in subsequent sections.

Our fruitful overall experience then made it possible to approach the intensities of the IR bands, in addition to their positions [49]. The values of the extinction coefficients of C≡C and C=C IR bands of ethyne and ethene, respectively, interacting with Cu^+^ or Ag^+^ in zeolites were determined on the basis of both quantitative IR experiments and quantum chemical DFT calculations using the QM/MM method. Both experimental and calculated values were in very good agreement, which further validated their reliability and encouraged the use of this type of modeling.

## 3. Dedicated QC Modeling of Metal Active Centers in Zeolites for Credible Description of Catalytic Properties

### 3.1. Concise Methodological Remarks

The majority of implementations of quantum chemical (QC) methodologies have been based, in principle, on one particle approximation in which the total energy and other observables of the N-electron system depend on the one-particle wavefunctions of N, called orbitals (or spinorbitals, if spin is included). QC computations became virtually effective for treating catalytic systems with the advent of the Kohn-Sham (KS) variant of density functional theory, based on one-electron functions. In practical implementations, these unknown orbitals are expanded as the linear combinations of certain known basis set functions; hence, the task of finding orbitals is reduced to finding the expansion coefficients in iterative procedures. Two main flavors of these basis set functions can be distinguished, namely: (i) square-integrable functions localized on atoms (usually Gaussian functions); and (ii) plane waves (PW), normalized within the box with periodic boundary conditions. For several (partially historical) reasons, the first class of functions is typical chosen by chemists, whereas solid-state physicists usually prefer the second type (although both periodic systems can be studied with atom localized functions and finite molecules with a PW basis). Sticking to PW basis sets, one would need an enormous number of basis functions to correctly represent the oscillating behavior of orbitals in the vicinity of nuclei. This problem can be overcome by introducing pseudopotentials (PP): the effective potentials replacing those due to nuclei and core electrons, which produce the same smooth tail of valence orbitals, above a certain distance from the nuclei. The introduction of PP reduces the cost of PW calculations, by diminishing both the number of PWs needed for orbital expansion and the number of electrons explicitly considered, at the price of neglecting electronic core states (at least in the original formulation of PP). The proper construction of PP is a nontrivial task, but currently, PP theory is well developed, and robust and fairly transferable PP datasets are available.

The computational cost of quantum mechanical calculations grows with the number of electrons (for localized atomic basis sets) or with the size of the unit cell (for PW basis sets). Unfortunately, zeolites, being low-density and frequently low-symmetry solids, usually fall into both of the above categories (e.g., hundreds of atoms/cell and cell volumes above 1000 Å^3^). This fact has for a long time hampered quantum mechanical calculations for the realistic extended models of zeolites, limiting the application of periodic DFT only to zeolites with small unit cells, like chabazite (T_12_O_24_ formula and cell volume about 800 Å^3^) [55]. Attempts to overcome this problem in the past involved either (i) switching to low-level/low-cost methods, like molecular mechanics (MM), applicable to realistic models of periodic zeolite lattices; or (ii) employing high-level/high-cost methods (DFT, quantum chemistry) for simplified cluster models, representing only finite fragment of zeolite lattice.

The electronic structure does not appear explicitly in the MM approach. Instead, the total energy of the studied systems (molecules or crystals) is approximated by the combination of assumed functions of atomic positions, called interatomic potentials functions (IPF) or force fields. These functions depend on interatomic distances (to capture two body interactions), angles and torsions (three- and four-body interactions), mixed terms, and so on. MM calculations are very cheap in comparison to QM methods, because the number of interacting particles is diminished by an order of ten or more (within MM ‘elementary particles’ are whole atoms or ions) and the analytical formula for energy is known from the beginning. Consequently, models consisting of millions of atoms or more are treatable at the MM level with modern computational resources. The disadvantage stems from the approximated nature of MM. The somewhat arbitrary choice of IPF can miss entire classes of physical properties, for example, representing two-body interactions using harmonic potential may predict reasonable equilibrium bond length, but cannot reproduce bond dissociation or the thermal expansion of solids. IPF depends on the number of parameters that must be fitted either to experimental data or to data computed from first principles for the test systems. IPF parametrized for a given set of observables (structures, elastic constants, vibrational frequencies, etc.) may poorly reproduce other quantities for the same class of chemical compounds. Furthermore, the transferability of IPFs and their parametrization between various systems is somewhat limited.

The compromise solution, which makes it possible to retain the relatively low computational cost of the cluster models and improve the latter through an approximate description of the extended environment, is the embedded cluster method. In this hybrid approach, the finite and rather small cluster is treated at a high level of theory, whereas the much larger (finite or periodic) environment is described at a more approximate—and less computationally demanding—low level of theory. If the high and low levels of theory represent a variant of the quantum mechanics method (DFT or one of the wavefunction-based methods) and MM, respectively, such hybrid approaches are referred to as the QM/MM method. The two main schemes for embedding clusters are the electrostatic and mechanical schemes. Electrostatic embedding relies on putting the QM cluster in the environment of the point charges (or higher multipoles) and adding the respective electrostatic terms to the cluster’s Hamiltonian. This approach may, however, overestimate the polarization of the cluster. In mechanical embedding, the geometry of the whole system is relaxed, with the QM cluster being subjected to forces arising from both itself and the environment, treated by a low-level method. Hence, the influence of the environment is indirect; no explicit terms are added to the cluster’s Hamiltonian, but the structures corresponding to the stationary points of energy (local minima or transition states) obtained in this way for the embedded cluster differ from those of the free cluster.

In our studies, we applied the variant of the embedded cluster method developed by Sauer and Sierka, referred to as the combined quantum mechanics-interatomic potential function approach (QMPot) [56,57]. Here, the zeolite lattice with periodic boundary conditions, described by MM, encapsulates the finite-size cluster, treated at the QM (usually DFT) level. The cut between the QM cluster and the MM part is done through the O_QM_–T_MM_ bond, and the dangling bonds at O_QM_ are saturated with artificial H atoms, called link atoms. The link atoms are not freely optimized, but are always kept along the O_QM_–T_MM_ line, at a fixed distance from adjacent OQ_M_ atoms. The total QMPot energy can be expressed as:

E_QMPot_ = E_MM_(Lattice) + E_QM_(Cluster) − E_MM_(Cluster) + E_QM,MM_(Interface) (1)

The last term contains the interaction energies between the inner and outer part, arising due to the presence of link atoms terminating the clusters. This term would be exactly zero, if no bonds were cut between the inner and outer part of the system (as in the case of solvated molecules, without covalent solvent–solute interactions), which is not our case, due to the aforementioned cluster construction. However, through the proper parametrization of the IPF used in the MM part (i.e., if the energy contributions due to the terminal H atoms in the MM and the QM ones mimic each other), this last term can be made to be close to zero, and subsequently neglected. This parametrization was developed by Sierka and Sauer [56], and will be briefly described later.

The forces acting on the atoms in the QMPot method, obtained by differentiation of (1), are given by the following formulae:

F_A_ = F_A_,_QM_(Cluster)+ F_A_,_MM_(Lattice) − F_A_,_QM_(Cluster), A∈QM Cluster F_B_ = F_B, MM_(Lattice), B∈MM (2)

where A and B denote the coordinates of atoms belonging to the QM cluster and the MM-only part, respectively. The link atoms, as mentioned, are kept along the line of the proper O_cluster_–T_MM_ bond. The second energy derivatives are also available in this approach. It is worth mentioning that mechanical embedding schemes like QMPot can be straightforwardly extended to QM/QM schemes, e.g., where the high-level method for the cluster is second-order Møller-Plesset perturbation theory, and the low-level method for the lattice is periodic DFT [58].

The advantage of the QMPot approach is that with a computational cost that is roughly equal to that of free cluster models, one obtains an approximate treatment of the periodic lattice. Because no robust methods of van der Waals treatment at the DFT level (like Grimme’s DFT-D [59]) were known at the time of QMPot development, an added bonus of QMPot was the possibility of including the dispersion interactions in at least the MM part of the calculations. QMPot has been proven to be particularly successful in the refinement of properties of catalytic centers in large unit cell zeolites, such as MFI [44,60], which even today remains quite demanding for periodic DFT (T_96_O_192_ unit cell formula). On the other hand, the proper choice of the QM cluster (its size and shape) remains a somewhat arbitrary issue. Both embedded and free clusters must be constructed in such a way as to avoid the interaction between ‘the meaningful’ atoms (like Cu(I) ions with adsorbed molecules, in our case) and the artificial H atoms terminating the cluster.

All reviewed QMPot results were obtained with the eponymous QMPOT program [56], which couples GULP [61] code for periodic MM calculations with the Turbomole suite for molecular QM modeling [62,63]. At the MM level, polarizable core–shell IPF are used, where lattice cations (Si, Al, H) are simply point charges, whereas lattice O atoms are represented by dipoles. This approach is particularly suitable for materials that are on the border between covalent and ionic solids, like zeolites. The careful parametrization of all atoms, including link H atoms, makes it possible to neglect the last term in Formula (1); this set of parameters was found by Sauer’s group by fitting IPF to the DFT data for cluster models [56,60]. For the atoms that are always within the QM cluster boundaries, like the adsorbed probe molecules considered in our work (e.g., NO or C_2_H_4_), where all the nearest neighbor covalent interactions were exclusively treated at QM level, the choice of parametrization was a less critical issue; therefore, IPF to approximate long-range electrostatic and van der Waals interactions was simply taken from the available datasets, like Universal Force Field [64]. At the DFT level, the Perdew-Burke-Ehrnzerhof (PBE) gradient functional [65] was applied, along with the triple zeta polarized basis sets, as a compromise method between the quality of data and computational cost. By crosschecking the PBE data with more accurate, but more expensive methods, it was concluded that PBE reliably predicted the geometries of the studied systems, although it frequently overestimated ligand-to-Cu(I) binding energies for the reasons discussed below. The adsorption energies presented in this work are ‘corrected PBE values’, i.e., scaled by the factors estimated from hybrid DFT or post-HF calculations for model systems (see source papers for details).

### 3.2. Selected Case Studies

In the following paragraphs, we will illustrate, on the basis of several examples, the process of building and gradual improvement of dedicated computational protocols, which serve then to capture several properties and processes on a particular zeolite. The QMPot studies discussed below describe in further detail our attempts to explicitly treat the zeolitic environment, and are focused on the faujasite (FAU)-type zeolite, which is known to exhibit several interesting catalytic and adsorption properties [66]. FAU is a large-pore (over 7 Å in diameter) and high-Al content zeolite; FAU with Si:Al ≈ 3 is called ‘zeolite Y’, and that with Si:Al ≈ 1 is called ‘zeolite X’. The idea to theoretically study this type of zeolite came from the collaborating experimental group of Prof. Jerzy Datka at Jagiellonian University, where the adsorption of probe molecules on Cu(I) exchange was studied by means of infrared spectroscopy [41,42,67]. One of the puzzling features of FAU is that despite having a much larger Cu content and larger pores, thus facilitating diffusion, it exhibits much lower activity in terms of NO decomposition than MFI zeolite [11]. The computational studies discussed below aimed to clarify the nature of different adsorption adducts observed spectroscopically in Cu(I)–FAU and shed some light on the differences in the reactivity of various zeolites.

To illustrate the complexity of modeling as a result of the diversity of possibilities in zeolitic structures, let us quote the first of the QMPOt studies on Cu(I)–FAU, co-authored by one of us, dealing with the CO adsorption of Cu(I)–FAU [68]. The models developed in this work were used as the starting points for the subsequent QMPOt studies on Cu(I)–FAU. For periodic MM calculations, the primitive rhombohedral unit cell of FAU, consisting of T_96_O_192_, was used. Only one Cu(I) ion per model was considered, and the remaining charge compensating cations (in models with more than a single Al atom) were protons. Two categories of FAU models were considered: (i) the high-silica models (HS), where 1–4 Al atoms were put in only the part of the FAU lattice forming the cluster at the QM level; and (ii) the Y model, where one-third of the T positions were substituted by Al atoms, as in real Y zeolite. The Al and H distribution in the latter followed the available experimental data [69,70]. The Al distribution in all models always obeys the Loewenstein rule, excluding Al–O–Al linkages. Unfortunately, no generally realistic model of zeolite X could be constructed within the QMPot approach, as with such high loading of Al and charge compensating extralattice cations, the interaction between the latter and the terminating cluster OH groups would be unavoidable and could lead to computational artifacts. Therefore, the conclusions presented below for zeolite X were tentatively derived from specific models of site II with the 3Al/6T ring (the highest local Al loading, possible only within X zeolite) and site III (less stable sites claimed on the basis of experimentation to be occupied by Cu(I) in zeolite X). The basic QM clusters used for modeling Cu(I) sites and their interactions with adsorbing molecules were built of 8 to 12 T atoms (T = Si or Al), but enlarged clusters (up to 22 T atoms) were also used in some cases, if necessary.

**Figure 2:**
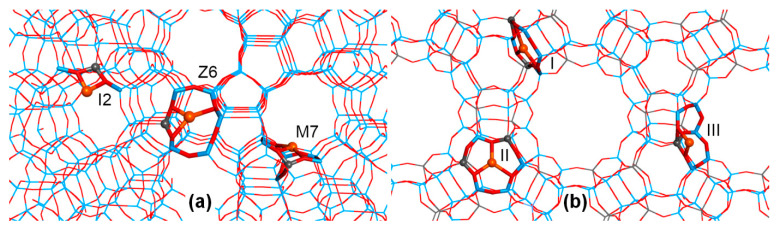
View of the lattices of zeolites (**a**) MFI and (**b**) FAU, with the main Cu(I) sites depicted (terminology for Cu(I) sites in MFI taken from Nachtigallová et al. 1999 [16]). The colors used for the atoms are: Cu—orange, Al—grey, Si—blue, O—red.

Three main positions were identified for extraframework cations in the FAU lattice (refer to Figure 2)—site I, within the hexagonal prism, site II, at about the middle of the 6T ring face of the cubooctahedral cage, and site III, above the 4T ring faces of the cage—with each of these sites having some distorted subspecies. The diffraction experiments for Cu(I)–Y zeolites indicated that over half of the Cu(I) ions were located in the base of the hexagonal prism (called I’), with the remainder located in site II and its variant, slightly displaced above the plane of 6T ring, called II’ [71]. The occupation of site III was not reported in Cu(I)–Y, though it cannot be excluded in Cu(I)–X at higher Cu/Al loadings. To evaluate the stability of these copper sites, Cu(I) binding energy was defined as the energy obtained through the dissociation of Cu(I)–FAU into the gas phase Cu(I) cation and the negatively charged lattice. Such binding energy is roughly equal for Cu(I) in positions I’ and II, and over 100 kJ/mol higher than that for site III. This is in agreement with experimental data showing the preferable occupation of sites I’ and II [71]. For site II, the increase in the number of Al atoms in the 6T ring (from 1 to 3) is associated with the moderate increase in Cu(I) binding energies (by about 20 kJ/mol per additional Al atom). Interestingly, 6T rings with two Al atoms in *meta-* and *para-* positions bind Cu(I) equally strongly. To describe Cu(I) interaction with lattice O atoms, we introduce the shorthand notation *m*(*n*), which indicates a Cu(I) ion bound to *m* lattice O atoms belonging to *n* distinct TO_4_ tetrahedra. The most stable I’ and II sites mostly have 3(3) coordination, whereas the less stable site III predominantly has twofold 2(1) or 2(2) coordination (see Figure 2). Threefold coordination of Cu(I) and mean Cu–O distances in the range 2.05–2.12 Å (depending on the model) predicted by QMPot are consistent with the available X-ray absorption spectroscopy data for Cu(I)–Y (with a Cu–O coordination number equal to 2.8 and a mean Cu–O distance of 1.99 Å) [71]. In all models, Cu(I) ions are located nearly in the plane of the 6T ring, which corresponds to site II in Ref. [71]. Site II’, displaced above the plane of the 6T ring, was not found in the QMPot models. This minor discrepancy could be due to the simplicity of the employed models (only a single Cu(I) ion per unit cell), but one cannot exclude that the error is related to refining the X-ray data (e.g., imposing some symmetry constraints).

**Figure 3:**
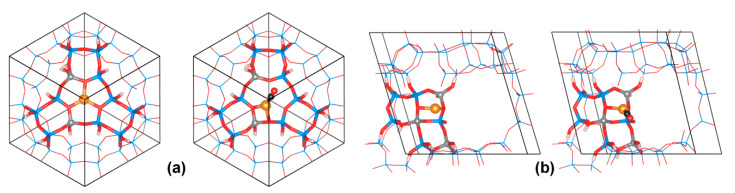
The optimized structures of the QMPot models used for the modeling of CO adsorption on Cu(I) exchanged FAU, the embedded cluster (QM level) depicted with balls and sticks, the remaining unit cell contents (MM level) with lines: (**a**) the models of site II (12T cluster), the equilibrium 3(3) Cu(I)–lattice coordination is switched to 2(1) upon CO adsorption; (**b**) site III model (8T cluster), here, the Cu(I) binding mode is 2(2) before and after CO adsorption. *m*(*n*) coordination denotes the Cu(I) ion bound to the *m* lattice O atoms belonging to *n* distinct tetrahedra. The following colors were used for the atoms: Cu—orange, Al—grey, Si—blue, O—red, H—white, C—black.

The next step of the QMPot study was the adsorption of CO on Cu(I) sites. The binding of the CO molecule via the C atom to Cu(I) ions at site II weakens the binding of the Cu(I) ion to the lattice, resulting in the displacement of Cu(I) ions above the plane of the 6T ring and switching the coordination from 3(3) to, usually, 2(1) (see Figure 3). CO adsorption on site III usually proceeds without significant changes in Cu–O coordination. Therefore, the total Cu(I) coordination upon CO binding remains trigonal, but with two bonds to the lattice O atoms and one to the C atom. Similar displacement of Cu(I) ions due to interaction with ligand molecules were found in previous QMPot studies on MFI and FER zeolites, and these were assigned to the increase of the Cu(I) radius due to electron donation from the ligand’s orbitals to the Cu(I) 4*s* state [44]. Additionally, XRD studies for Cu(I)–Y reported that CO adsorption was associated with the increase of Cu(I) ions in more exposed site II’ positions [71].

The experiment showed two monocarbonyl bands in Cu(I)–FAU (2140 and 2160 cm^−1^ in Cu(I)–Y, 2130 and 2155 cm^−1^ in Cu(I)–X), with one band clearly blue-shifted with respect to the gas phase CO stretching frequency (2143 cm^−1^) [67]. DFT cluster modeling is known to have problems with the description of the CO stretching frequency in Cu(I) monocarbonyls, always predicting the red-shift [21]. This topic was analyzed in depth in Nachtigall’s group, where two main sources of problem were identified: (i) overestimated backdonation from Cu 3*d* to CO π* states; and (ii) lack of polarization of the CO molecule by the opposite wall of zeolite in small clusters [72]. The first issue can be partially improved by using hybrid functionals, which shift the energy of the LUMO in the CO molecule up; to solve the second issue, it is necessary to use larger cluster models. In this work, the scaling method based on the linear relationship between CO bond length and CO stretching frequency was proposed as the most efficient method for accurately computing the latter quantity. This approach, when applied to the discussed QMPot FAU models, enabled us to identify the low and high frequencies in Cu(I)–Y monocarbonyl bands as stemming from Cu(I) site II with 2 and 1 Al atoms/6T ring, respectively [71]. For Cu(I)–X, the low-frequency band is likely due to site II with a 3Al/6T ring, and the high-frequency band is likely due to site III. For site II CO vibrations calculated with enlarged and ‘regular’ clusters are nearly the same, for site III, the blue-shift increased by about 10 cm^−1^ with increasing cluster size. Apparently, in the more confined site II, the Cu(I)–ligand interactions were predominantly moderated by the local Al content, while for the more exposed site III, the polarization effect by the lattice was more prominent. The influence of ‘from bottom’ and ‘from top’ effects on CO vibrations in zeolites was analyzed in depth by Nachtigallová et al. [73].

CO binding energies usually show the reverse trend to Cu(I) binding energies. The QMPot CO binding energies calculated at the QMPot level for site II with the 1-2Al/6T ring (60–75 kJ/mol) were in excellent agreement with the reported microcalorimetric data for Cu(I)–Y (65–80 kJ/mol) [74] when hybrid B3LYP was applied (or the PBE data were accordingly scaled). The original PBE values were nearly twice as large, which can be attributed to the overestimation of backdonation by gradient functionals. Site II with the 3Al/6T ring binds CO slightly more weakly (50 kJ/mol), whereas the CO binding energies for site III are in the range of 100–120 kJ/mol. Generally, the stronger the ligand adsorption is, the smaller the rearrangement of Cu(I) siting induced by ligand attachment is.

**Figure 4:**
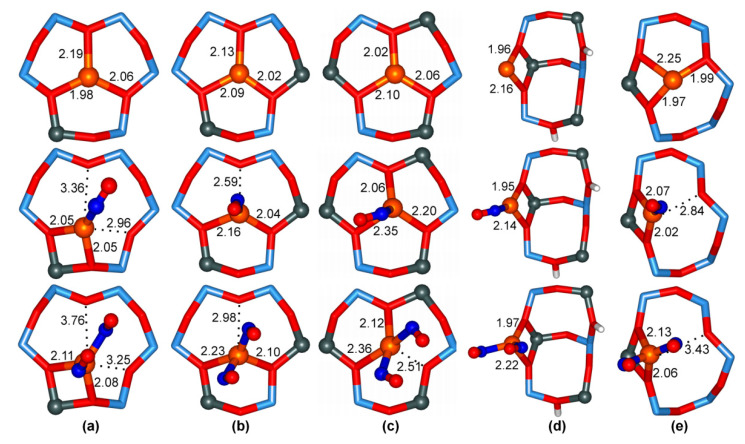
The Cu(I) siting and its change upon adsorption of one and two NO molecules, according to QMPot modeling (only the nearest neighborhood of Cu(I) ions shown, all distances in Å): (**a**–**c**) site II in FAU with 1, 2 and 3 Al/6T rings, (**d**) site III in FAU and (**e**) Z6 site in MFI. The colors used for the atoms are: Cu—orange, Al—grey, Si—blue, O—red, H—white, N—dark blue.

The next study we performed was combined IR spectroscopic and computational work on the adsorption of NO on Cu(I)–FAU, with an attempt to assign the entire NO spectrum [75]. Both mono and dinitrosyl species were considered. Single NO adsorption was found to displace Cu(I) ions from the 6T ring, although this was somewhat weaker than in the case of CO. Apart from the stable mononitrosyls with 2(1) Cu(I) coordination, the second local minimum was usually found in the same 6T ring, with 2(2) Cu(I) binding mode, or even with 3(3) coordination preserved in the 3Al/6T ring (see Figure 4). PBE again overestimates the adsorption energies by nearly double with respect to B3LYP, with the latter being reported to correctly reproduce the experimental NO heat of adsorption in Cu(I)–MFI [44]. The corrected QMPot/PBE binding energies for site II are in the range of 30–50 kJ/mol (the largest values are for the 1Al/6T ring). NO adsorption on coordinatively unsaturated site III does not displace Cu(I) ions, and the ligand binding energies are much higher (90–110 kJ/mol), comparable to the values reported for MFI zeolite [44].

Several types of dinitrosyl species were considered, and singlet species with both ligands attached to Cu(I) ion by N atoms and with weak intermolecular bonds via O atoms were always found to be the most stable (see Figure 4). Unlike in the case of mononitrosyls, B3LYP was found to predict no binding of the second NO molecules (i.e., adsorption energy was nearly 0, or even endothermic), which is in striking contradiction to the experiment. Test calculation performed at the CASPT2 level (single-point calculation at DFT geometry of a single octahedron T1 cluster model) showed that the hybrid B3LYP functional failed to deal with the case of rather strong static correlation in dinitrosyl species, whereas gradient PBE behaved qualitatively correctly (although the energy values were overestimated by nearly double, as in the case of mononitrosyls). This issue was studied in depth using correlated wavefunction techniques [76], and will be discussed in subsequent sections of this paper. The QMPot/PBE binding energies for a second NO, corrected with respect to CASPT2 models, were quite similar for all considered Cu(I) models, not only for FAU, but also for the additionally tested models of Cu(I) site In MFI. This is understandable, as the addition of the second NO molecule to mononitrosyl does not displace the Cu(I) ion any further (i.e., the 2(1) or 2(2) coordination is preserved). This shows that the determining step in the direct decomposition of NO over Cu(I)–zeolites is the formation of mononitrosyl species. In consequence, the stable Cu(I) site In FAU 6T rings adsorb NO about two to three times more weakly than more loosely bound Cu(I) ions in MFI, and thus FAU, despite its much higher Cu loading, exhibits lower activity in the direct NO decomposition than MFI.

Unlike the case of CO adsorption, the measured NO stretching modes in Cu(I)–FAU are always red-shifted, and the dinitrosyls start forming nearly simultaneously with mononitrosyls. For Cu(I) nitrosyls, DFT predicts the frequency red-shift correctly [21]. As Cu(I) mononitrosyls have a strongly bent structure (Cu–N–O angle of about 140°), the coupling between bending and stretching modes is more prominent, and it is unlikely that the linear bond length-frequency relationship would hold. Therefore, we decided to stick to harmonic DFT frequencies, as they are sufficient for at least the qualitative analysis in this case. With respect to mononitrosyl stretching frequencies, we observed a decrease with increasing numbers of Al atoms in the 6T ring, but, unlike in the CO case, they were also dependent on Cu–lattice binding, namely, for a given *n*Al/6T ring, mononitrosyls with Cu(I) in 2(1) coordination would have higher NO stretching modes than those with 2(2) (or 3(3) in 3Al/6T ring) binding. The highest NO stretching frequencies were numerically predicted for site III. However, no such frequencies were experimentally registered in high alumina zeolite X, although site III may become populated by Cu(I) ions at higher Cu loadings. This could be due to the somewhat easier formation of dinitrosyl species at these sites and the partial overlapping of mononitrosyl modes at site III with symmetric dinitrosyl vibrations at site II. Dinitrosyl stretchings were found by us to be less site specific and thus less predictive, due to the partial overlap of dinitrosyl bands calculated for various models. The detailed assignment of experimental nitrosyl bands based on QMPot models is given in Table 1.

More insight into the interaction between the ligand and Cu(I)–zeolite was achieved in our subsequent study on the adsorption of ethene, where we coupled QMPot modeling with charge transfer analysis, as described in the next part of this review ([77], see Section 4). We found that for ethene, the shift in C=C stretching frequency was dependent only on the Cu(I) ion coordination to the lattice, i.e., in adsorption complexes with 2(2) copper coordination, this vibrational mode was higher than in these with 2(1) copper coordination. The difference between C=C bands obtained from harmonic DFT analysis (about 10 cm^−1^) matched very well the observed split between such bands in Cu(I)–FAU [42]. The QMPot calculations for Cu(I)–MFI predicted a single C=C band, which was in agreement with the experimental data [42], as the final Cu(I) attachment to the lattice was of the 2(1) type in all considered models. The ETS-NOCV analysis showed that (i) for the neutral (C_2_H_4_/Cu(I)–zeolite) subsystems, π* backdonation (weakening C=C bond) was larger for adducts with 2(1) Cu(I) binding, and (ii) for the charged (C_2_H_4_-Cu^+^/zeolite^−^) fragments, more efficient electron flow from the zeolite lattice (acting as an electron sink) to the adsorption adduct was seen for the 2(1) coordination case, as well. Unlike the previously studied cases, the PBE adsorption energies for ethene at site II are in very good agreement with the available microcalorimetric data for Cu(I)–Y [74], while the B3LYP values seem to be underestimated, possibly due to the worse description of some weaker interactions (e.g., σ* backdonation from Cu 3*d*_z_^2^ orbital or weak overlapping between Cu orbitals and C-H bonds orbitals) by the latter functional.

In short, our QM/MM results showed that there was no single rule governing frequency shifts of probe molecules adsorbed in Cu(I) exchanged zeolites. Depending on the details of adsorbate–adsorbent interactions, it can be moderated by (i) the local geometry of the adsorption site, (ii) the local Al loading, and (iii) final Cu–O_lattice_ coordination upon adsorption. We are aware that the above studies were limited to models consisting of isolated Cu(I) sites; hence, the issue of the interaction of single adsorbate molecules with multiple Cu(I) sites (very likely for Cu(I)–X at high Cu/Al concentration) is still open (note that the presence of carbonyl complexes with a CO molecule bridging two extralattice cations in zeolites was shown on the basis of combined theoretical and experimental studies by Garrone et al. [78]). Gradient functionals are usually sufficient to obtain a qualitatively correct description of the studied systems, although for quantitative agreement with the experimental data one usually needs to apply some corrections derived from more advanced computational methods. Although some results obtained in the reviewed QMPot modeling of Cu(I)–FAU had been reported in earlier free cluster studies (e.g., Cu(I) displacement induced by CO adsorption) [79,80,81], the systematic analysis of issues like local Al loading, the presence of multiple local minima for energy for a given active site, or obtaining the adsorption energies of experimental quality is only possible when the extended environment of the zeolitic active site is considered in at least an approximate manner, as in the QM/MM approach.

Finally, it should be noted that in recent years, the application of periodic DFT has become standard in zeolite modeling, due to both the improving performance and the decreasing prices of hardware and the progress in theory and in scientific software development. Notably, the prevalence of ultrasoft PP by Vanderbilt [82], and the subsequent development of the projector augmented waves approach by Blöchl [83] (the former can be seen as a special case of the latter, more general, method) made it possible to speed up the periodic DFT calculations. In these approaches, because they represent core states in radial grids, far fewer (about one third) PWs are required to expand the valence states than with traditional PP, while not decreasing the quality of computational results.

**Figure 5:**
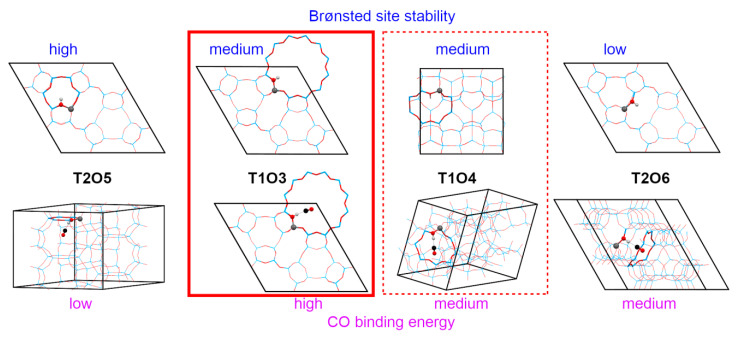
CO adsorption on Brønsted site In MAZ zeolites, as predicted by periodic DFT modeling. T*x*O*y* denotes Brønsted acid sites with Al occupying the T*x* crystallographic position and the protonated O atom situated in y crystallographic sites (the MAZ lattice with distinct T and O crystallographic positions is shown in the upper insert). The formation of stable carbonyls is likely due to Brønsted sites compromising their intrinsic stability and the strength of CO binding. The proposed assignment of experimental OH bands is as follows: (1) the more red-shifted band is due to the more stable carbonyls in the large 12T cavity (solid red frame), and (2) the less red-shifted band is due to the less stable carbonyls in the more confined 8T cavity (dotted red frame). The colors for the atoms are: Al—grey, Si—blue, O—red, H—white, C—black.

As an example of periodic DFT studies on zeolites, below, we discuss our recent paper on the acid form of mazzite (MAZ) zeolite, which was (to the best of our knowledge) the first example of periodic DFT study on this zeolite [84]. The motivation for this study was an experimental report on the observation of an unusually large red-shift of OH stretching frequencies upon CO adsorption [85], with the latter value frequently being taken as the measure of Brønsted acidity in zeolites (see, however, [86] for a critical discussion of the determination of acidity using this method). We used a doubled hexagonal MAZ cell along the *c* direction and put a single Al^3+^/H^+^ pair in this model; hence, the stoichiometry was HSi_71_AlO_144_. There is a total of seven possible Al^3+^/H^+^ pairs owing to the topology of MAZ and the number of distinct crystallographic positions of atoms (two for T and four for O atoms, with H^+^ always sitting at the O atom adjacent to Al). We optimized the structure for the H-MAZ systems, and then for the models with the CO molecule adsorbed onto the Brønsted sites. Instead of performing harmonic vibrational analysis, which could be very costly for such large unit cells, while still not being exact, the stretching OH frequencies were approximated in the same was as for the CO case, i.e., using the linear scaling method based on OH bond length, as proposed by Nachtigall et al. [87]. The advantage of this approach is that it also includes the correction for anharmonicity. Accurate anharmonic frequencies can be obtained on the basis of molecular dynamics calculations, which will be briefly described in the final sections; however, such calculations are very expensive. The results of DFT modeling for H-MAZ are summarized in Table 2 and Figure 5. The stability of monocarbonyl adducts stems from the interplay of the low distortion of the Brønsted site during CO adsorption and steric hindrance, which prevents the energetically preferred almost linear H–C–O arrangement. We were able to identify two distinct experimental OH bands (characterized by bigger and smaller OH stretching red-shifts) as corresponding to Brønsted site In the wider 12T and narrower 8T cavities, respectively.

## 4. Electronic Dialogue between the Ligand, Metal Active Center and a Zeolite Framework

With the development of reliable models of catalytic active sites at the molecular level, novel tools arrived, serving to extract relevant information from electron density and its deformation imposed by specific interactions in the multi-component system consisting of the zeolite framework, the catalytic center and the substrate. The contribution of this type of analysis to the knowledge regarding catalytic processes will be discussed in more detail in this section for the case of ethene activation by copper and silver cationic site in zeolite, discussed already in Section 2 and Section 3.

To shed light on the origin of the weakening of the carbon–carbon bond upon interaction with transition metal centers in zeolites, the charge and energy decomposition scheme ETS-NOCV [88] was applied, as implemented in the ADF package [89,90]. This approach also makes it possible to understand metal–ligand bonding in terms of qualitative and quantitative delineation of donation ligand→metal and backdonation metal→ligand charge transfer channels, as it decomposes total interaction energy (ΔE_total_) into physically meaningful contributions: ΔE_total_ = ΔE_elstat_ + ΔE_disp_ + ΔE_Pauli_ + ΔE_orb_. The first term represents the classical electrostatic interaction between the selected fragments in the system’s geometry, which is often attributed to ionic features. The second contribution, ΔE_disp_, corresponds to the semi-empirical Van der Waals component developed by Grimme [91]. The next term, ΔE_Pauli_, concerns Pauli repulsion between occupied orbitals of fragments. Finally, the orbital interaction ΔE_orb_ term, covers donation/backdonation contributions, as well as intra-fragment polarizations due to mixing between occupied molecular orbitals of one fragment with virtual orbitals of the second fragment, and vice versa. The NOCV method enables diagonalization of the differential density matrix into contributions corresponding to relevant chemical bonding channels: $\Delta \rho_{\mathrm{orb}}\;=\;\sum_{i}\Delta \rho_{\mathrm{orb}}\left(i\right)$. To this end, the combined ETS-NOCV makes it possible to obtain Δρ_orb_(*i*) contributions (donation, backdonation, etc.), as well as the corresponding energies ΔE_orb_(*i*) for any system, even without symmetry [88]. Such extracted bonding channels formed between a metal active center and a ligand are useful for in-depth understanding of the origin of IR red-shift in stretching CC frequencies due to metal–ligand bonding. It has also been shown that ETS-NOCV is suitable for the reliable description of various non-covalent interactions [92,93].

**Figure 6:**
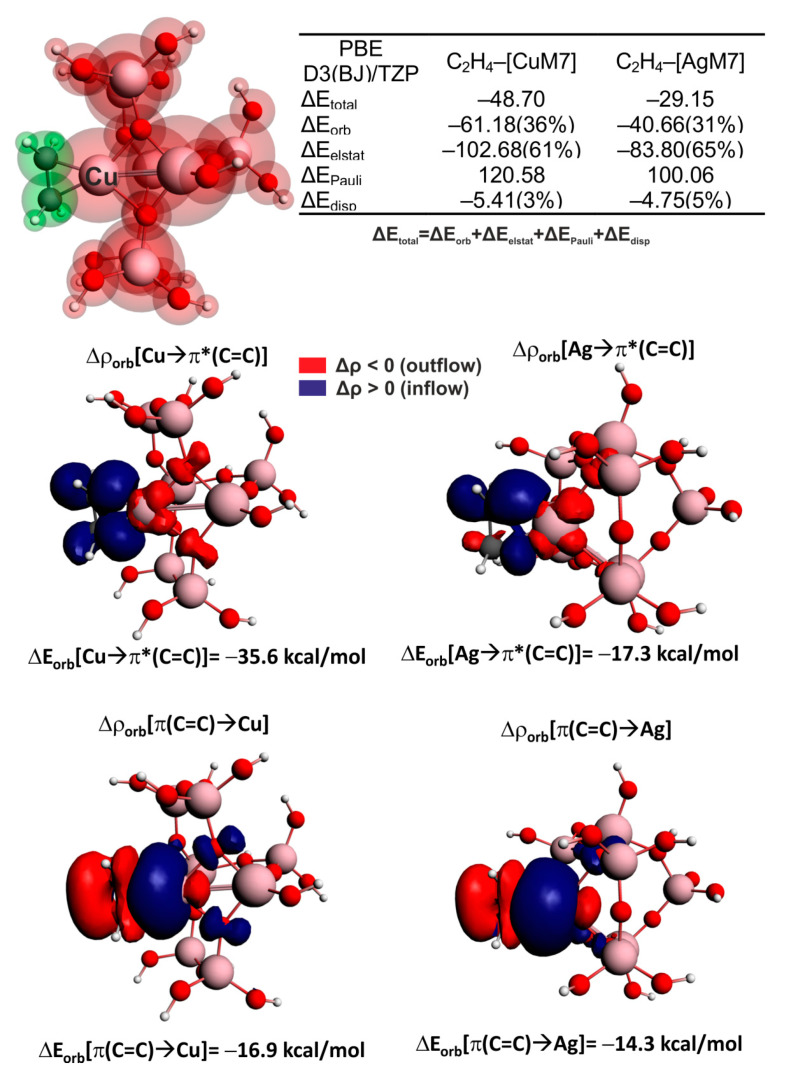
The results of ETS-NOCV/PBE-D3(BJ)/ZORA/TZP energy decomposition describing [C_2_H_4_]–[Cu/AgM7] bonding. Additionally, the most relevant NOCV-based deformation density channels Δρ_orb_(*i*), together with the corresponding energies, ΔE_orb_(*i*) are presented.

The cluster model depicted in Figure 6 (top, left) was applied, containing three constituent components: the zeolite surrounding (M7, the cluster composed of two fused T-O-T rings [33]), the metal active center (Cu or Ag), and ethene. It can be seen from the inset in Figure 6 that the overall interaction energy between ethene and the copper-containing fragment, [C_2_H_4_]–[CuM7] (ΔE_total_=−48.70 kcal/mol), is far more pronounced than that for the corresponding [C_2_H_4_]–[AgM7] bonding (ΔE_total_=−29.15 kcal/mol). This is qualitatively in agreement with experimental observations suggesting that copper sites exhibit better activating power of multiple carbon–carbon bonds in hydrocarbons than silver sites. London dispersion contribution is found to be the least quantitatively important (it covers only 3–5% of the overall stabilization ΔE_orb_+ΔE_elstat_+ΔE_disp_) and is similar for both metals (Figure 6). The electrostatic/ionic (ΔE_elstat_) component is the most crucial at an absolute level, since it covers ~61–65% of the overall stabilization, whereas the orbital interaction (ΔE_orb_) is also significant, and encompasses 31–36% (Figure 6). It can be further explained that stronger bonding of [C_2_H_4_]–[CuM7] vs. [C_2_H_4_]–[AgM7] is rooted in both more efficient electrostatic (|ΔE_elstat_|, by 18.9 kcal/mol) and charge delocalization (|ΔE_orb_|, by 20.5 kcal/mol) constituents (Figure 6). Subsequent NOCV-based analyses made it possible to conclude that the π-backdonation from copper to the empty π*(C=C) is roughly twice as efficient with respect to the corresponding Ag→π*(C=C) transfer (ΔE_orb_[Cu→π*(C=C)] = −35.6 kcal/mol vs. ΔE_orb_[Ag→π*(C=C)]= −17.3 kcal/mol). This provides a nice rationale for the larger C=C red-shift at Cu/ZSM-5 vs. Ag/ZSM5 (by |Δν_exp_| = 44 cm^−1^), described in the second section.

**Figure 7:**
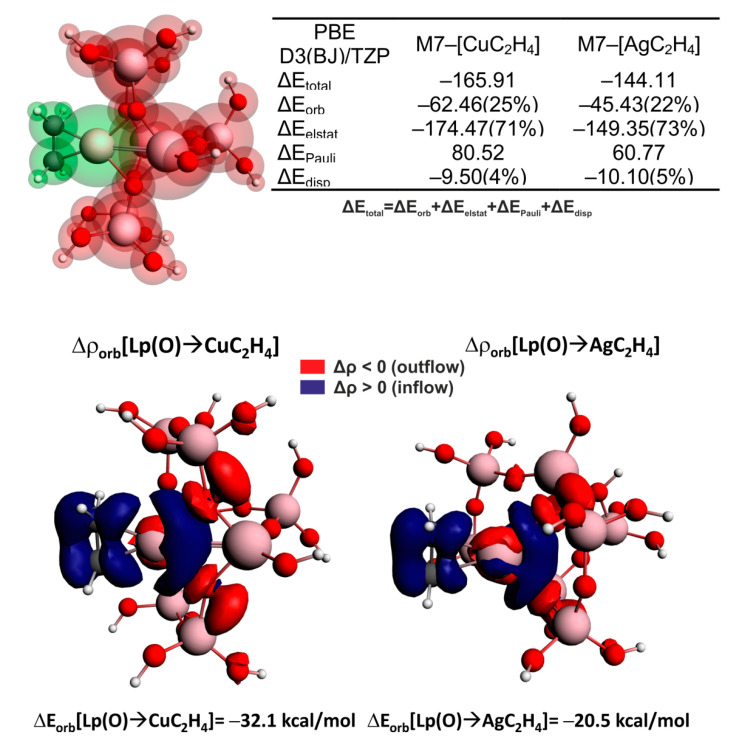
The results of ETS-NOCV/PBE-D3(BJ)/ZORA/TZP energy decomposition describing [M7]–[Cu/AgC_2_H_4_] bonding. Additionally, the most relevant NOCV-based deformation density channels Δρ_orb_(1+2), together with the corresponding energies ΔE_orb_(1+2), are presented.

Interestingly, the opposite charge delocalization, described as Δρ_orb_(π(C=C)→metal), is roughly similar for both Cu and Ag centers, being quantitatively inferior over Cu/Ag→π*(C=C) (Figure 6, bottom). Hence, the more efficient C=C activation by Cu/ZSM-5 vs. Ag/ZSM-5 may predominantly be traced back to a more pronounced π-backdonation activation channel for Cu→π*(C=C) than for Ag→π*(C=C). It is intriguing to note that closer inspection of the contour Δρ_orb_[Cu→π*(C=C)] reveals additional electron outflow from the lone pairs of ZSM5 oxygen atoms that is not present in the corresponding contour Δρ_orb_[Ag→π*(C=C)]. To examine in more detail the role of zeolite surroundings (represented by the M7 cluster) in the bonding between C_2_H_4_ and the embedded cation, an alternative fragmentation pattern was considered in which [C_2_H_4_(Cu/Ag)]^+^ and the zeolite [M7]^−^ were the two fragments forming the bond. The nature of this bonding was examined within the [M7]–[C_2_H_4_Cu/Ag] partition scheme on the basis of ETS-NOCV analysis, and the outcomes are gathered in Figure 7.

First of all, the M7 fragment interacts more efficiently with [CuC_2_H_4_] than [AgC_2_H_4_] (|ΔE_total_| changes by 21.8 kcal/mol), which, in turn, is related to weaker stabilization stemming from both ionic (|ΔE_elstat_| by 25.12 kcal/mol) and charge delocalization (|ΔE_orb_| by 17.03 kcal/mol) constituents (Figure 7, top). As far as the most relevant charge delocalization channels Δρ_orb_ [Lp(O)→Cu/AgC_2_H_4_] are concerned, the outflow of electron density from the lone electron pairs of the framework oxygen atoms toward the metal centers, as well as ethylene, can be clearly seen. This proves the important role of zeolite in supplying electrons to metal centers and, accordingly, amplifying the π-backdonation charge transfer processes, Cu/Ag→π*(C=C). It is important to highlight that such electron pumping from the adjacent oxygen atoms is far more effective for Cu/ZSM5 than for Ag/ZSM5; note, ΔE_orb_[Lp(O)→CuC_2_H_4_] = −32.1 kcal/mol vs. ΔE_orb_[Lp(O)→AgC_2_H_4_] = −20.5 kcal/mol (Figure 7).

**Figure 8:**
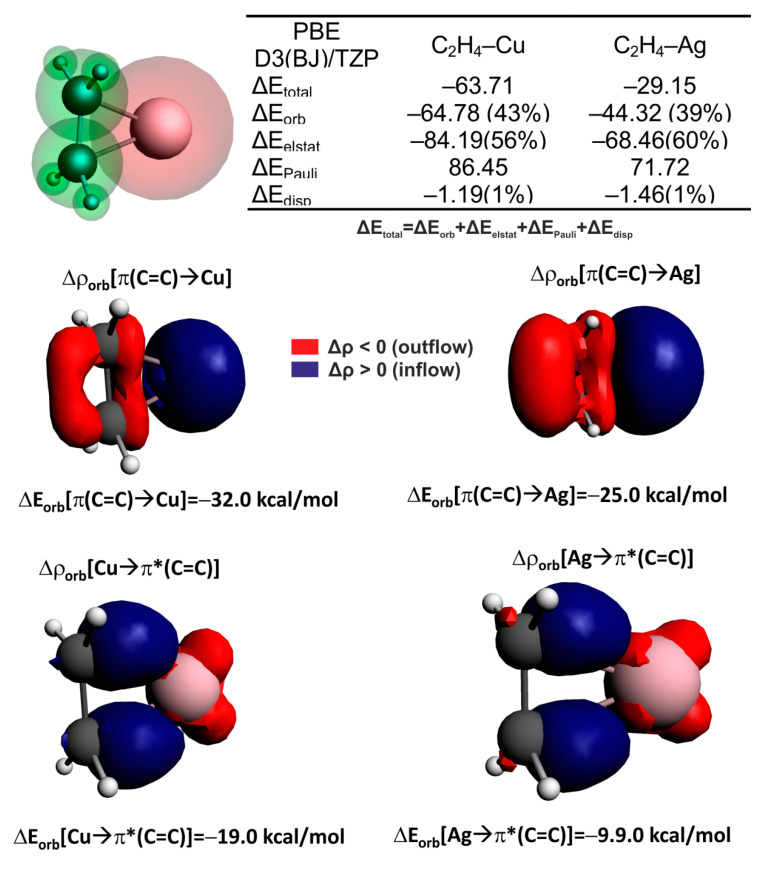
The results of ETS-NOCV/PBE-D3(BJ)/ZORA/TZP energy decomposition describing C_2_H_4_–Cu/Ag bonding. Additionally, the most relevant NOCV-based deformation density channels Δρ_orb_(*i*), together with their corresponding energies ΔE_orb_(*i*), are presented.

To confirm the crucial role of the zeolite framework as an electron reservoir promoting the most important C=C activation channel, Cu/Ag→π*(C=C), it is very useful to finally consider the reduced bonding situation, C_2_H_4_–Cu/Ag, where the zeolite part (M7) is omitted. It is important to point out that without the surrounding framework, significant deterioration of the π-backdonation Cu/Ag→π*(C=C) is observed: the corresponding contributions are diminished to −19.0 kcal/mol for Cu–C_2_H_4_ and −9.9 kcal/mol for Ag–C_2_H_4_, respectively (Figure 6), in line with [94]. Accordingly, the opposite charge transfer Δρ_orb_(π(C=C)→metal) overcomes the metal-to-ligand charge transfer Cu/Ag→π*(C=C) for the bare C_2_H_4_–Cu/Ag system (Figure 8). This last observation fully contradicts the case of C_2_H_4_–[Cu/AgM7] bonding depicted in Figure 8. These outcomes reconfirm the critical role of the zeolite framework, which has been found to act as an electron pool promoting π-backdonation Cu/Ag→π*(C=C) channels, and, most importantly, activating the C=C bond (Figure 6). Copper is found to be a better π-electron donor/mediator (to anti-bonding π*(C=C)) than silver—this could be related to the relatively smaller size of the former, which facilitates very efficient simultaneous interaction with hydrocarbons, as well as the zeolite environment. Similar observations were also found to be valid for the activation of ethyne [95] and formaldehyde over ZSM5 [96] as well as for ethene adsorption on MFI [77].

All these findings clearly indicate that electronic factors, in this case the type and efficiency of charge flow channels between the active site (acting as Lewis or Bronsted center) and the reactant, play crucial roles in the catalytic process. Moreover, quantifying the importance of individual channels using NOCV analysis made it possible to pinpoint subtle differences in the activity of various cations and distinct zeolites towards specified substrates.

## 5. Transition Metal Ions: The Challenge for Electronic Structure Calculation and Interpretation

Zeolites embedding transition metal ions are receiving considerable attention owing to their unique catalytic properties, notably in environmental catalysis [97,98,99]. However, in terms of computational modeling, the presence of TMI site in a material presents numerous challenges related to the variability of the metal oxidation states, the possible existence of multiple spin states, the significant interplay between electronic and geometric degrees of freedom, and the strong electron correlation effects affecting the electronic structure of the TMI (d electrons) and the metal–ligand interaction [100,101,102].

The importance of correlation effects in TMI-containing systems calls for their adequate treatment both at the level of electronic structure determination and during geometry optimization. DFT methods are widely used owing to their favorable compromise between computational efficiency and accuracy. Indeed, modern DFT methods are able to reliably predict molecular geometries, and they approach “chemical accuracy” (~5 kJ mol^−1^) when calculating relative energies, as evidenced in numerous benchmark studies [100,103,104].

However, certain energetic properties of TMI sites with potential relevance in catalysis, such as metal–ligand bond energies and spin-state splittings, are known to be computationally much more problematic [105,106,107,108]. For such challenging electronic properties, not only are the computed results strongly dependent on the choice of functional (with discrepancies of ~50 kJ mol^−1^ not being rare), but the optimal choice of functional is also system dependent (in particular, it is dependent on the TMI oxidation state and its coordination environment, including ligand field strength or binding mode), so that finding functionals that perform universally well for different systems is currently problematic [109,110,111,112]. This also means that attention is required when extrapolating the conclusions of benchmark studies in the literature to other systems, not included in testing set [100]. For instance, with regard to spin-state splittings, quantitative benchmark studies are not numerous, and when they refer to experimental data, these are often data for isolated TMIs in gas phase [113] or of spin-crossover complexes with organic ligands [114], which have significantly different coordination environments from those of TMIs in zeolites.

The controversies surrounding the accuracy of DFT energetics have stimulated interest in using correlated wave function theory (WFT) methods, in particular (1) single-reference CCSD(T), or other coupled cluster methods, and (2) multireference methods based on the complete active space (CASSCF) approach, such as CASPT2, RASPT2 or NEVPT2 (see reviews in Refs. [100,102,115,116], and the references therein, to find more details, including explanation of the methods’ acronyms).

None of the WFT methods can compete with DFT in terms of computational efficiency or user friendliness; additionally, none of them can be considered a replacement for DFT methods in the efficient exploration of the potential energy surface (e.g., geometry optimization with vibrational analysis, identification of possible mechanistic steps) or in periodic calculations for crystal models. However, the WFT methods can be very useful for calibrating the DFT predictions and helping to choose the best-performing functional. Such benchmark studies are usually carried out as single-point energy calculations for simplified cluster models whose geometries are optimized at the DFT level. For example, Pulido and Nachtigall applied the CCSD(T) method to simplified cluster models (T1-type, built of a single aluminum-oxygen tetrahedron) of zeolitic Cu(I) site In order to calibrate the DFT predictions of relative stabilities for Cu-nitrosyl and dinitrosyl species with relevance to the mechanism of deNOx processes [76]. Following this direction, we studied the energetics of these mono- and dinitrosyl species in even greater detail, and included not only single-reference CCSD(T), but also multireference methods (CASPT2 and RASPT2) [117]. Another recent example of applying multireference calculations to zeolite models is the study of Vogiatzis et al., where CASPT2 and RASPT2 calculations were used to clarify the electronic structure and spin-state splittings of a [Cu_3_(μ-O)_3_]^2+^ cluster inside the pore of MOR zeolite and to rationalize which of the three available O atoms was most reactive towards methane monooxygenation [118]. Advanced WFT calculations can be also be used to predict spectroscopic features of TMI site In their various possible forms (including hypothetical ones), with the aim of checking which species is most consistent with the registered experimental spectrum. For example, Snyder et al. employed a multireference CASSCF/CASPT2 approach to characterize the spectroscopic features of Fe-zeolite cluster models, providing a significant contribution toward the full characterization of the Fe active sites responsible for the catalytic hydroxylation of methane [119].

There is occasionally a concern as to whether DFT and single-reference correlation methods are capable of describing the electronic structure of TMI sites qualitatively correctly. This probably stems from the widespread, but (in the authors’ opinion) mostly anecdotal association of all transition metal molecules with multireference problems [100,120,121]. Indeed, multireference methods are indispensable for dealing with certain electronic states of isolated transition metal ions or atoms and their small molecules (e.g., diatomics); they should also be used to properly handle electronic degeneracy in certain excited states, which may be of relevance for computational spectroscopy. However, in low-symmetry environments, such as in zeolite frameworks, electronic degeneracy is usually lifted, meaning that the above problem is of little practical relevance. Moreover, even in the presence of electronic degeneracy, a single-determinant method can still represent (at least qualitatively correctly) a single component of the degenerate state [112]. Even more complicated electronic structures, such as antiferromagnetically coupled multi-center TMI complexes, can be described reasonably well using spin-polarized (unrestricted) DFT, leading to broken-symmetry (BS) solutions (see Refs. [120,122] for a review). However, when using this approach, one should be aware of the spin contamination artefact, and that the resulting spin density is nonphysical (see below for a recent example in the context of zeolite catalysis).

The fact that single-determinant methods, including DFT, can provide (for many interesting cases) a qualitatively correct description of the electronic structure of TMI sites *does not* mean that their predictions are automatically expected to be quantitatively accurate. The above-mentioned problem with predicting spin-state energetics is a good example. Although the majority of low-energy spin states for mononuclear TMI complexes *can be* reasonably described by a single Slater determinant without significant spin contamination, the obtained DFT results are highly dependent on the functional, and are thus potentially inconclusive (see above). Although the problems of approximate DFT methods in accurately describing spin-state energetics may appear to be rooted in the treatment of exchange interactions, they have more to do with imperfect coverage of the correlation effects underlying the metal–ligand bonds [123,124].

It should be noted that accurate description of spin-state energetics is important for properly characterizing the catalytic properties of TMI sites, because different spin states not only have different magnetic properties, they also have different structural features (higher spin states typically have weaker metal–ligand bonds due to the occupation of antibonding orbitals), and this may also lead to differences in their chemical reactivity [125,126]. If the ground spin state of the reactant is different from that of the product, the spin-state energetics will indirectly contribute to the reaction energy. If this spin-state conversion contribution is not reproduced quantitatively accurately (which is probable if DFT methods are used in the modeling), there might be a significant error in the computed reaction energy, even if all the reactants and products, including their spin states, appear to be described qualitatively correctly [127].

The accurate prediction of spin-state energetics is a challenge not only for DFT, but also for WFT methods. In the study of zeolitic Co^2+^ sites with ammonia co-ligands interacting with NO [128,129], we found that the resulting mononitrosyl species could exist in singlet or triplet spin states, predicted (at least by some methods used) to be very close to one another in terms of energy. Interestingly enough, the two alternative spin states were found to differ remarkably in their ability to activate the N–O bond (see below for more details). Alas, the singlet–triplet splitting was remarkably challenging to accurately compute, with sizable discrepancies not only between different DFT methods, but also between WFT ones. In particular, for model complexes [Co(T1)(NH_3_)_2_(NO)]^+^ (where T1 = [Al(OH)_4_]^−^ served as a simple model of the lattice) and [Co(NH_3_)_5_(NO)]^2+^, the CASPT2 and CCSD(T) results differ from each other by ~20 kcal/mol [125]. By comparison with the experimentally characterized salt containing the same [Co(NH_3_)_5_(NO)]^2+^ cation, it was experimentally concluded that the pentaamine cation was in a low-spin ground state, which was in agreement with the CCSD(T) results, but not with the CASPT2 ones. This conclusion might appear unexpected in view of the presumed multireference character of TMIs (and especially those containing the nitrosyl ligand). However, the benchmarking results for several different TMI complexes, where quantitative experimental data of spin-state energetics are available, have so far confirmed the high accuracy of the CCSD(T) method in predicting spin-state splittings [111,112]. This is parallel to other known examples in which the CCSD(T) method was able to correctly describe the structure and energetics of transition metal systems [121], for instance the bond energies in transition metal diatomics [130]. More benchmarking is needed for the problem of spin-state splittings to establish definite conclusions, but for relatively simple mononuclear TMI species, where suitable reference data can be derived from the experimental data, the performance of CCSD(T) is appealing, especially when contrasted with the much greater errors obtained not only with DFT methods, but even with such “respected” methods as CASPT2, NEVPT2 and multireference configuration interaction when using the standard active space [111,112]. The systematic bias of CASPT2 in favor of higher spin states is partly mitigated [111,112] by the CASPT2/CC approach of Phung et al., i.e., the CASPT2 description of valence correlation combined with the CCSD(T) description of metal outer-core correlation [131].

While the canonical CCSD(T) method is too computationally expensive to be widely used in the modeling of materials, it has become more affordable owing to availability of computationally more efficient, locally correlated implementations, such as the DLPNO-CCSD(T) method developed by Neese et al. (see Ref. [132] and references therein). For main group chemistry, both closed-shell and open-shell systems, the DLPNO approximation is known to lead to negligible errors only [132], and thus DLPNO-CCSD(T) results can be treated as a benchmark that is practically equivalent to the canonical CCSD(T). In the context of acidic zeolite catalysis, Plessow and Studt used the DLPNO-CCSD(T) results for several catalytically relevant reaction energies and barriers with the aim of benchmarking the DFT methods [133]. However, in the context of the spin-state energetics of open-shell TMI sites, there is contradictory evidence regarding the accuracy of the DLPNO approximation [134,135].

In addition to providing quantitatively correct energetics, another computational challenge with TMIs is to describe their interaction with redox-active and open-shell molecules, in particular nitric oxide (NO) and oxygen (O_2_). Whereas the binding of closed-shell ligands is qualitatively well explained in terms of the Chatt–Duncanson model ([136], see also Section 4), NO and O_2_ are non-innocent ligands, and actively participate in the electronic structure by sharing their unpaired electrons (one in the case of NO, two in the case of O_2_) with the d electrons of the TMI. The resulting electron delocalization is usually described using Enemark-Feltham notation, e.g., {M-(NO)*_k_*}*^n+k^*, where the superscript *n*+*k* denotes the sum of the *n* valence d electrons of the TMI and *k* electrons contributed by *k* NO ligands (the analogous notation is used for complexes with O_2_ ligand, keeping in mind that O_2_ contributes two electrons). Due to the electron delocalization in such complexes, the oxidation states of the metal and ligand are unclear, and should usually be considered fractional (“non-innocent ligand”).

Because zeolites exchanged with certain TMIs (Cu^I^, Co^II^) are efficient NO decomposition catalysts [97,98], there has been considerable interest directed toward understanding the electronic factors responsible for the activation of the N–O bond in adducts with zeolitic TMI sites. One way of addressing this problem is to use the NOCV method (natural orbitals for chemical valence, introduced in Section 4), which can identify and quantify the electron-flow channels accompanying metal–ligand bond formation.

The NOCV approach, as discussed previously in Section 4, has been very successful in the case of closed-shell ligands. In the case of open-shell ligands, such as NO, the analysis has to be carried out in a spin-resolved (SR) way, i.e., separately for the α and β spin manifolds, leading to the SR-NOCV generalization. A given electron flow channel may appear in only one spin manifold or in both spin manifolds; in the latter case, the spin-resolved contributions should be added (taking care to note whether they describe the electron flow in the same or in the opposite direction) in order to characterize the global importance of the channel. Moreover, when constructing the promolecule for SR-NOCV analysis, not only the fragments’ geometries, but also their electronic states should match those in the metal–ligand complex being formed [129].

The latter requirement is particularly important for the setup of the NO promolecular fragment, which is a necessary prerequisite for the SR-NOCV analysis of bonding in metal–nitrosyl complexes. For isolated NO molecules, due to their cylindrical symmetry, their unpaired electron is able to equivalently occupy any combination of the degenerate π*_x_ and π*_y_ molecular orbitals. However, when NO is considered to be the promolecular fragment for the SR-NOCV analysis, the unpaired electron has to be assigned to one of these π*-type orbitals in the way that is most consistent with the electronic distribution in the resulting nitrosyl complex (i.e., to minimize the redundant deformation density accompanying the metal–NO bond formation) [137,138]. Failure to meet this condition would result in the appearance of artificial electronic flows on the NO fragment that do not contribute to the description of metal–ligand bonding, but noticeably complicate the obtained results and their interpretation. The construction of a suitable promolecule (i.e., avoiding the above artifacts) can be guided by the inspection of molecular spin density distribution [137,139], whereas in more complicated cases, the analysis of BS-DFT or CASSCF natural orbitals can be used to properly assign the fragment’s electronic states [129]. For nitrosyl complexes possessing bent metal–N–O units, the construction of promolecules is typically straightforward: the unpaired electron of the NO fragment should be placed in the π* orbital that has the maximum amplitude in the metal–N–O plane, whereas the choice of spin states for the NO and metal fragments is determined by the global spin state of the complex [137]. The situation is more complicated for nitrosyl complexes possessing linear metal–N–O groups [129,138]. In such complexes, the electronic density/spin density of the nitrosyl group has nearly cylindrical symmetry, making the decision as to which of the degenerate NO π* orbitals should be occupied in the promolecular fragment somewhat arbitrary. Accordingly, the NOCV results for such linear nitrosyls reveal the electronic flow channels whose only purpose is to restore the nearly cylindrical symmetry of the electronic density, but have little effect on the redistribution of the electron density between the metal and NO fragments [138]. It would appear attractive to employ a promolecule in which the two NO π* orbitals are half-occupied (i.e., the statistical ensemble rather than pure quantum state) for such cases, but unfortunately this would violate the basic assumption in the NOCV analysis that all orbitals occupied in the molecule and promolecule have identical occupation numbers (i.e., 1 in the SR version or 2 in the closed-shell case), because the so-called eigenvalue-pairing property of the NOCVs critically relies on that [140].

In a series of papers, we employed the SR-NOCV method to characterize NO bonding in zeolitic TMI sites of increasingly complex electronic structure, from the simplest Cu(I) site (closed-shell, d^10^ configuration) [139], through Cu(II) (open-shell d^9^) [137], up to the most complicated Co(II) (open-shell d^7^) [128,129] sites. In a related paper [138], we also used this methodology to study the bonding of NO to complicated Fe^II^ (open-shell d^6^) complexes with various ligands (although not serving directly as zeolite-type models). The studies of zeolitic Cu(I)/Cu(II) sites [137,139] made it possible to distinguish the following electron flow channels:

1. Backdonation from Cu to the empty NO π*
2. Donation of an unpaired electron from the occupied NO π* to Cu
3. Covalent Cu–NO contribution
4. Donation from the NO σ lone-pair into Cu

Please note that electron flows along channels (1) and (2) have mutually opposite effects in terms of N–O bond activation, because they lead to either an increase (1) or a decrease (2) in the electron population, with NO π* orbitals having an antibonding character with respect to the N–O bond. The relative importance of these channels, as quantified by their SR-NOCV eigenvalues, is remarkably different when comparing Cu(I) sites with Cu(II) sites. Backdonation (1) was shown to be the principal channel in the case of zeolite-Cu^I^ sites (observed in both the α and β spin manifolds); its high relevance is consistent with the weakening of the N–O bond, which was experimentally confirmed on the basis of IR spectroscopy as a red-shift of the NO vibration [139]. Comparison with the bare Cu^+^ ion, on which the N–O bond was experimentally deactivated (blue-shift of the NO vibration, lack of deNOx reactivity) revealed the crucial role of the zeolite environment: it is responsible not only for increasing the importance of the backdonation channel (1), but also for closing the unpaired electron donation channel (2), which is the principal channel for bare Cu^+^–NO. Interestingly, the simplest T1 and a much more elaborate model of zeolite lattice (M7) gave almost identical results in SR-NOCV analysis, suggesting that the fine details of the zeolite structure are not crucial for understanding the N–O activation mechanism, as long as the simplified model is able to correctly reproduce the TMI–zeolite coordination mode, which is certainly the case for Cu(I) sitting preferentially on alumina sites and adopting two-fold coordination with their lattice oxygens [139].

In the case of zeolite-Cu(II) sites, the NO ligand is bound more strongly (in energy terms) to the metal than in the case of analogous Cu(I) sites, but it is deactivated due to the diminished backdonation (2) and increased donation of the NO unpaired electron (1) [137]. Remarkable similarity was observed between the electron flow channels of (T1)Cu(II)–NO cluster and those of bare Cu^+^–NO. It could be postulated that the zeolite environment partly neutralizes the high charge of the embedded Cu^2+^ ion, so that its electronic properties are similar to those of isolated Cu^+^. An importance difference, however, between the two situations is that the acceptor orbital for channel (1) is no longer the Cu 4s orbital (a low-energy orbital in free Cu^+^, and a high-energy orbital if the ion is coordinated), but rather the unoccupied 3d orbital of Cu(II) [137].

Other studies in this series were focused on understanding the activation of N–O bonds by zeolitic Co(II) site In the presence of ammonia co-ligands [128,129]. Based on the results of IR spectroscopy and periodic DFT calculations, we constructed appropriate cluster models for the interaction between Co(II) sites and NO containing no, two, three, or five NH_3_ ligands. In the last case, the actual complex studied was [Co(NH_3_)_5_(NO)]^2+^, i.e., the Co(II) ion was no longer covalently bound to the zeolite lattice. For other complexes, the zeolite lattice was modeled using a single aluminum tetrahedron (T1). In the absence of NH_3_ co-ligands, two additional H_2_O molecules were added to mimic the coordination mode of Co(II) ion found in more complete zeolite models.

For the discussed Co–NO complexes, the results of SR-NOCV analysis confirmed the chief role of ammonia co-ligands in promoting the N–O bond activation, which is evidenced by the considerable red-shift of the NO vibration due to the lowering of the N–O force constant. Among the studied cluster models, the strongest activation of the N–O bond was predicted for [Co(T1)(NH_3_)_2_(NO)]^+^ and [Co(NH_3_)_5_(NO)]^2+^, both in the singlet state. The main electron-flow channels, as revealed by the SR-NOCV analysis, are backdonation from Co to NO (described in terms of NOCV orbitals perpendicular to the Co-N–O plane) and the spin-coupling channel related to the formation of the σ-type Co–NO bond (in the Co-N–O plane) [129]. Interestingly, the bond activation effect (as described both by the SR-NOCV metrics and the red-shift of NO stretching) is predicted for the complex with only three NH_3_ ligands, [Co(T1)(NH_3_)_2_(NO)]^+^, rather than for the complex with five such ligands, [Co(NH_3_)_5_(NO)]^2+^ [129]. This finding is at first sight counterintuitive, because NH_3_ is the stronger electron donor than the zeolite lattice. However, the observed trend can be rationalized by inspecting the geometries of these complexes. In the pentaamine complex, the presence of strong NH_3_ ligands exerts a strong trans effect on the Co-NO distance—thus leading to reduction of the electronic interactions responsible for the N–O bond activation—compared with the complex that possesses only three NH_3_ co-ligands, where the trans position is occupied by the weaker O-based ligand of the lattice [128].

Another observation made with regard to the discussed zeolitic Co-NO sites was that not only the number of NH_3_ co-ligands, but also the spin state of the resulting complex is relevant for the degree of N–O bond activation. In particular, for both [Co(NH_3_)_5_(NO)]^2+^ and [Co(T1)(NH_3_)_2_(NO)]^+^ complexes, the red-shift of the N–O stretching vibration was predicted to be much larger for the singlet than for the triplet state. This finding was corroborated by the NOCV analysis of the electronic interaction between the ammonia co-ligands and the rest of the respective complex, showing that indeed the flow of electrons from the NH_3_ donors towards the NO π* orbitals is more effective in the singlet than in the triplet state [128].

**Figure 9:**
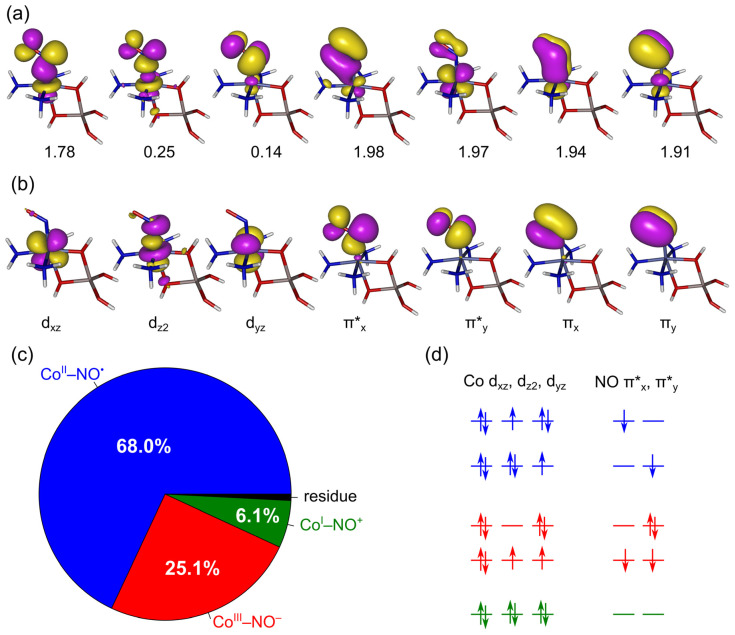
Typical results of CASSCF-VB analysis on the example of [(T1)Cu(NH_3_)_3_(NO)]^+^, singlet state (see Ref. [129] for details): (**a**) selected CASSCF(16,14) natural orbitals (delocalized), being linear combinations of the Co 3d and NO π*,π with fractional occupation numbers annotated below; (**b**) selected localized orbitals obtained by unitary transformation of the CASSCF(16,14) natural orbitals along with their qualitative designation as Co d_xz_, d_z2_, d_yz_ and NO π*_x,y_, π_x,y_ fragment orbitals; (**c**) percentage contributions of the participating resonance structures; (**d**) schematic representation of the most relevant individual electronic configurations in terms of the Co d_xz_, d_z2_, d_yz_ and NO π*_x_, π*_y_ fragment orbitals (colors correspond to colors used in the pie chart in (**c**)).

An alternative approach for understanding the charge distribution in metal–nitrosyl species is the valence bond analysis of the multiconfigurational CASSCF wave function, the so-called CASSCF-VB method [129,141,142]. In this approach, the CASSCF calculations are performed first (with suitable active space, which must comprise the bonding and antibonding orbitals describing the metal–NO bond, as well as all other orbitals that are relevant to describing the correlation effects in specific systems). In the next step, the active orbitals are localized on the desired fragments, such as (in the case discussed) the NO fragment and the rest of the complex. (Please note that the procedure of orbital localization and “reading” the resulting wave function expansion was considerably improved in Ref. [129] compared with earlier works). Since the CASSCF wave function is invariant to unitary transformations of the active space, the transformation from delocalized (natural or canonical) to localized active orbitals *does not* affect the wave function (and hence any molecular properties, including energy), but it may be highly desirable to simplify the interpretation. In particular, when active orbitals can plausibly be assigned to either the metal or the NO fragment, it is possible to interpret the CASSCF wave function as the quantum superposition of electronic configurations with well-defined charges on both interacting fragments, i.e., as resonance structures such as Co^II^–NO^0^, Co^III^–NO^−^, and Co^I^–NO^+^, and estimate their contributions to the total wave function (see the example in Figure 9). The results may be treated as descriptors of the N–O character in different coordination environments.

The discussed resonance structures are not to be confused with the formal and simplistic assignment of nitrosyl ligand in complexes as either NO^−^ or NO^+^, depending on the geometry of metal–N–O Typical results motif (the linear geometry is indicative of a NO^+^ character, the bent one of a NO^−^ character). The results of the CASSCF-VB analysis show that the electronic structure of a nitrosyl complex is best described as a superposition of different resonance structures, reflecting a covalency of the metal–NO bond. Even if, for some complexes, one type of resonance structure dominates, it seldom has a contribution as great as 90%. Moreover, it is possible the dominant resonance structure obtained by CASSCF-VB analysis is in disagreement with simplistic geometry-based assignment; for instance, Tomson et al. characterized Co–NO and Ni–NO complexes with almost linear metal–N–O motif as having a predominantly Co^II^/Ni^II^–NO^−^ electronic character [142].

In Ref. [129], the CASSCF-VB analysis was applied to the Co-NO complexes with ammonia co-ligands discussed above. All these complexes belong the Enemark-Feltham class {CoNO}^8^, so the participating resonance structures were Co^II^–NO^0^, Co^III^–NO^−^, and Co^I^–NO^+^, with negligible contributions from other possible ones (Co^0^–NO^2+^, Co^IV^–NO^2–^). The detailed analysis put forward in Ref. [129] confirmed that relative shares of these resonance structures correlated well, at least qualitatively, with other descriptors of the N–O bond strength: the stretching frequency and the force constant. In particular, the noticeable weakening of the N–O bond in the cluster model [Co(T1)(NH_3_)_2_(NO)]^+^, especially in the singlet state (see above), was also fully confirmed using the CASSCF-VB method, and this was ascribed to the most pronounced contribution of the Co^III^–NO^−^ resonance structure (i.e., the one having one more electron in the antibonding π* shell compared with free NO) and the smallest contribution of the Co^I^–NO^+^ resonance structure (i.e., the one having one electron less in π*) [129]. The CASSCF-VB method was shown to work reasonably well for nitrosyls with both bent and linear Co-N–O groups. This is a significant advantage over the SR-NOCV methodology, which is pushed to the limits with linear nitrosyls due to the requirement to (arbitrarily) select the fragment occupations in the promolecule, as discussed above.

Another recent application of the CASSCF-VB method in the context of zeolites is the study of Pietrzyk et al., where this approach was used to characterize the mononitrosyl {NiNO}^10^ adduct relevant to the mechanism of the selective catalytic reduction of NO with ethene over Ni/ZSM-5 zeolite [143]. The CASSCF-VB analysis for the electronic structure of the mononitrosyl complex revealed a dominant contribution (87%) of the antiferromagnetically coupled Ni^II^–NO^0^ resonance structure. There are additional contributions from the Ni^I^ –NO^+^ and Ni^III^ –NO^−^ structures, of which the first one was found to be the most important, which is consistent with the slight blue-shift of the NO vibration observed in the IR. Interestingly, although the leading resonance structure has an NO^0^ character, i.e., free a radical on NO group, only a small part of the spin density is confined to the NO group, which is in accordance with the interpretation of the HYSCORE spectroscopic results [143]. This is because the unpaired electron on NO is antiferromagnetically coupled to one of the Ni unpaired electrons, and thus only the second unpaired electron (on nonbonding d orbital of nickel) effectively contributes to the spin density. While the multiconfigurational CASSCF wave function can describe such antiferromagnetically coupled electronic structures properly, at the DFT level, the same has to be described with a BS solution. This BS solution has strong spin polarization, with a positive spin density on Ni and a significant negative one on NO fragments, making it very different from the CASSCF spin density or the one inferred from the HYSCORE experiment. Here, one should bear in mind that the BS-DFT solution is not expected to reproduce the physical spin density (i.e., that which is “probed” experimentally in magnetic resonance spectroscopies and obtained from multiconfigurational calculations). The spin polarization observed in such calculations is merely a way of mimicking the correlation effects (related to metal–nitrosyl bonding) by DFT [115,120,141] and, in particular, reflects the fact that NO predominantly has a mono-radical character. The actual spin populations appearing on the NO group in nitrosyl complexes are also known to be very sensitive to the choice of functional [141]. One should thus be careful when interpreting the spin densities obtained from BS-DFT calculations, even if the predicted structures and energies are very often quite correct.

## 6. The Latest in Modern Molecular Modeling

Our main idea behind conducting the present review was to show how experiment and theory have accompanied each other in researching new areas, in this case, zeolites in catalysis. We commenced the review with early simplistic modeling of straightforward properties of zeolitic active sites to assist the interpretation of experimental research triggered by the discovery of exceptional activity of copper(I) site in Y and ZSM-5 zeolites towards NO decomposition. The next section illustrated how the process of building and gradually improving dedicated computational protocols made it possible to explicitly treat the zeolitic environment, and thus to capture several properties and processes as being dependent on zeolite type and active site location.

Parallel to the development of reliable computational models of catalytic active sites at the molecular level, novel tools were designed, either serving to extract hidden information from electron density and its deformation upon specific interactions in a multi-component molecular system, or to increase the accuracy of electronic structure calculations. Section 4 and Section 5 focused on the reciprocal relation between the physicochemical processes in question (e.g., bond breaking or forming in ligands) and the fine details of the electronic structure of the active site. Section 4 was devoted to the rationalization of activating ability of cationic metal sites in zeolites, stemming from electron density flows between the zeolite framework, the cationic center and the substrate. In conjunction with smart model design, quantifying the importance of individual electron density flow channels made it possible to pinpoint subtle differences in the activity of various cations and distinct zeolites towards specified substrates. Section 5 dealt with electronic phenomena related to the variability of the metal oxidation states and the possible existence of multiple spin states, affecting the electronic structure of the TMI sites and metal–ligand interaction. Critical discussion regarding the growing accuracy and applicability of advanced quantum chemical methods illustrated the quest to interpret modern experiments. Finally, the material presented in Section 5 nicely documented the process of shrinking the gap between the two areas of research on the basis of their mutual inspiration.

When addressing the future of molecular modeling, some remarks should also be given regarding the computational determination of reactive free-energy surfaces, stressing the credibility of predictions and qualitative insights into heterogeneous catalysis [5,144]. For example, the density functional theory molecular dynamics (DFT-MD) provides an efficient framework for accurately computing several types of properties and spectra [145,146]. The major benefits of DFT-MD approaches lie in the ability to naturally take into account the effects of temperature and anharmonicity. Thus, DFT-MD methods can be used to study large and complex systems, such as peptides, DNA strands, amorphous solids, and molecules at surfaces or in solution in finite temperatures [147]. However, one should be aware of the limitations of the classical dynamics methods in describing vibrational progressions in electronic spectra [148].

Our preliminary experience with respect to ab initio molecular dynamics (AIMD) simulations made it possible to introduce the explicit temperature into the description of the CO adsorption at acid site in faujazite [149], and to extract the anharmonic vibrational frequencies from MD trajectories under ambient conditions for NO sorbed onto cobalt zeolite catalyst in excess of ammonia [129,150]. In the first case, we were able to calculate for the first time the vibrational frequencies for CO adsorbed on Brønsted active site in faujazite at T = 100 K. The novelty of our approach comes down not only to good reproduction of the experiment conducted at T = 100 K, but also to showing that the CO adsorption was stable only at low temperatures, whereas MD simulations at T above 273 K did not indicate the existence of stable complexes. This agrees very well with IR experiment, able to register the spectrum characteristic for CO bound to zeolite surface solely in the low-temperature regime. On the contrary, QC-only (T = 0) optimization would probably yield some forms of weakly bound complexes, depending on the calculation parameters.

In the case of NO adsorption on Co-exchanged zeolite in the presence of ammonia, MD simulations at T = 293K allowed us to reproduce the dynamic behavior of the system traced by IR measurements under ambient conditions, depending on ammonia content [129,146]. A similar approach was used by van Speybroeck et al. to study the status of copper cations for Cu-CHA catalysts in contact with reactants and intermediates under realistic operating conditions in the selective catalytic reduction of nitrogen oxides with ammonia [147]. Our simulations reproduced the measured NO spectra and made it possible to assign individual IR bands to various forms of [Co(II)(NH_3_)_n_(NO)] complexes in the zeolite environment and to attribute specific values of the NO red-shift to the predominance of either Co^II^–NO^0^, Co^III^–NO^−^ or Co^I^–NO^+^ resonance structures in the total wave function. The latter were taken as the descriptor of the N–O character in different coordination environments and served to rationalize the activation/deactivation of the NO bond by means of the donating ability of the cobalt site, moderated by both the zeolite environment and by the ammonia co-ligand (see also Section 5).

The last two examples nicely illustrate the main motif of the present review with respect to the mutual benefits stemming from fruitful cooperation between theoretical and experimental studies. They show also that deep insights into the complex reality of catalysis would not be possible without the joint efforts of the researchers involved either in fine laboratory measurements or in the development and smart applications of modern quantum chemical methodology.

## Figures and Tables

**Table 1:** The QMPot harmonic frequencies (ω_QMPot_) calculated for various models of Cu(I) nitrosyls in FAU zeolite (X and Y subtypes), and the proposed assignment of experimental frequencies (ν_exp_) [75].

Zeolite	Cu(I) Siting	Mononitrosyls				Dinitrosyls ^1^	
		CN_Cu_	ω_QMPot_ (cm^−1^)	ν_exp_ (cm^−1^)	CN_Cu_	ω_QMPot_ (cm^−1^)	ν_exp_ (cm^−1^)
Y	II–1Al/6T	2(1)	1790	1815	2(1)	1695, 1820	1730, 1825
		2(2)	1780	1793	2(2)		
	II–2Al/6T	2(1)	1760–1775		2(1)	1680, 1810	
		2(2)	1755–1775	1780	2(2)		
X	II–3Al/6T	2(1)	1760	1772	2(1)	1675, 1800	1700, 1820
		3(3)	1735	1757	3(3)	1665, 1790	
	III	2(1), 2(2)	1800		2(1)	1695, 1820	

^1^ Antisymmetric and symmetric band. For clarity only mean QMPot values for a given class of models are given.

**Table 2:** The selected properties of Brønsted site In MAZ, bare and with adsorbed CO molecules, as predicted by periodic DFT calculations: relative energetic stability (ΔE), OH bond lengths (R_OH_), scaled frequencies (ν_OH_) and the value of red-shift upon CO adsorption (Δν_OH_), CO binding energy (E_CObinding_) and HCO angle (Θ_HCO_). The sites assigned to the experimentally observed more and less red-shifted OH bands (with the experimental Δν_OH_ being −370 and −270 cm^−1^, respectively) are double and single underlined, respectively.

Brønsted Site	ΔE (kJ/mol)	R_OH_//ν_OH_ (Å//cm^−1^)	R_OH(+CO)_//ν_OH_ (Å//cm^−1^)	Δν_OH_ (cm^−1^)	E_CObinding_ (kJ/mol)	Θ_HCO_ (°)
$\underline{\underline{T1O1}}$	$\underline{\underline{9}}$	$\underline{\underline{0.9783//3593}}$	$\underline{\underline{1.0054//3236}}$	$\underline{\underline{-357}}$	$\underline{\underline{-28}}$	$\underline{\underline{174.2}}$
T1O2	6	0.9791//3593	1.0118//3151	−430	−13	167.4
$\underline{\underline{T1O3}}$	$\underline{\underline{4}}$	$\underline{\underline{0.9783//3593}}$	$\underline{\underline{1.0053//3237}}$	$\underline{\underline{-356}}$	$\underline{\underline{-27}}$	$\underline{\underline{174.6}}$
T1O4	9	0.9792//3581	1.0030//3267	−314	−20	171.8
T2O2	4	0.9802//3568	1.0099//3177	−391	−9	165.1
T2O5	0	09808//3560	0.9981//3332	−228	−12	177.0
T2O6	31	09836//3523	1.0139//3124	−399	−22	179.8
